# Influence of Melatonin on Cerebrovascular Proinflammatory Mediators Expression and Oxidative Stress Following Subarachnoid Hemorrhage in Rabbits

**DOI:** 10.1155/2009/426346

**Published:** 2010-01-31

**Authors:** Qi Fang, Gang Chen, Weiwei Zhu, Wanli Dong, Zhong Wang

**Affiliations:** ^1^Department of Neurology, The First Affiliated Hospital of Soochow University, Suzhou 215006, China; ^2^Department of Neurosurgery, The First Affiliated Hospital of Soochow University, Suzhou 215006, China

## Abstract

The aim of this study is to analyze whether melatonin administration influenced the nuclear factor-kappa B (NF-*κ*B) activity, proinflammatory cytokines expression, and oxidative response in the basilar artery after SAH. A total of 48 rabbits were randomly divided into four groups: control group, SAH group, SAH + vehicle group, and SAH + melatonin group. All SAH animals were subjected to injection of autologous blood into cisterna magna twice on day 0 and day 2. The melatonin was administered intraperitoneally at a dose of 5 mg/kg/12 h simultaneously with SAH from day 0 to day 5. The basilar arteries were extracted on day 5 after SAH. As a result, we found that vascular inflammation and oxidative stress were induced in all SAH animals. In animals given melatonin, basilar arterial NF-*κ*B and pro-inflammatory cytokines were decreased in comparison to vehicle-treated animals. Measures of oxidative stress also showed significant downregulation after melatonin treatment. Furthermore, administration of melatonin prevented vasospasm on day 5 following SAH. In conclusion, post-SAH melatonin administration may attenuate inflammatory response and oxidative stress in the spasmodic artery, and this may be one mechanism involved in the therapeutic effect of melatonin on the subsequent vasospasm after SAH.

## 1. Introduction

Cerebral vasospasm is the most common cause of disability and death in patients suffering from aneurysmal subarachnoid hemorrhage (SAH) [1]. Cerebral vasospasm is commonly caused by the presence of blood products, especially oxyhemoglobin (OxyHb) in the subarachnoid space. Even though the trigger role of OxyHb in the developing vasospasm is well known, the exact mechanism(s) of vasospasm is still unknown. The possible mechanisms may include: upregulated vascular inflammation, endothelial apoptosis, increased free radical products and oxidative stress, adenosine diphosphate- (ADP-) induced vasomotor changes, inhibition of nitric oxide (NO) pathway, protein kinase C (PKC) pathway in vascular smooth muscle, among which inflammation and oxidative stress both play important roles in the pathological process of vasospasm [2–4].

Recently, several studies on experimental models of SAH have demonstrated that melatonin can prevent vasospasm [5–7]; however, the exact mechanism of vasoprotective effect is still unknown. As mentioned by Ersahin et al., treatment with melatonin due to its free radical scavenging properties significantly inhibited SAH-induced lipid peroxidation and neutrophil infiltration of the brain tissue on the second day of SAH induction in rats. However till now, no study was found in the literature to investigate the effects of melatonin on vascular inflammatory response and oxidative stress after SAH. 

There is increasing evidence that inflammation is critical in the development of cerebral vasospasm [8]. Nuclear factor-*κ*B (NF-*κ*B) and proinflammatory cytokines, such as tumor necrosis factor *α* (TNF-*α*), interleukin (IL)-1*β*, and IL-6, may be key factors contributing to the inflammation in spasmodic arteries [9]. Oxidative stress is a state of imbalance between free radical production, particularly the reactive oxygen species (ROS) leading to progressive oxidative damage, and the ability of the organism to defend itself against them. Usually, ROS can be efficiently scavenged by the antioxidant defense system such as superoxide dismutase (SOD) and glutathione peroxidase(GSH-Px). Malondialdehyde (MDA) is the end product of the major reactions leading to significant oxidation of polyunsaturated fatty acids in cellular membranes, and thus it serves as a reliable marker of oxidative stress [10]. Thus, the aim of the current study was to determine whether melatonin could regulate the level of inflammatory agents and biomarkers of oxidative stress in the basilar artery after experimental SAH in rabbits.

## 2. Materials and Methods

### 2.1. Animals

The animal use and care protocols, including all operation procedures, were approved by the Animal Care and Use Committee of Soochow University and conformed to the Guide for the Care and Use of Laboratory Animals by the National Institute of Health. Forty-eight adult male New Zealand White (NZW) rabbits weighing from 2.2 to 2.8 kg were purchased from the Animal Center of the Chinese Academy of Sciences (Shanghai, China). They were acclimated in a humidified room and maintained on the standard pellet diet at the Animal Center of Soochow University for 10 days before the experiment. The temperature in both the feeding room and the operation room was maintained at about 25°C.

### 2.2. Two-Hemorrhage Rabbit Model

Experimental SAH was produced according to our previous study [11]. The rabbits were anesthetized with an intramuscular injection of a mixture of ketamine (25 mg/kg) and droperidol (1.0 mg/kg) on day 0. Under spontaneous breathing, a 23-gauge butterfly needle was inserted percutaneously into the cisterna magna. After withdrawal of 1.5 mL CSF, the same amount of nonheparinized fresh autologous auricular arterial blood was slowly injected into the cisterna magna for 1 minute under aseptic technique. Then animals were kept in a 30° head-down position for 30 minutes, which made blood flow from the cisterna magna to the basilar cistern more easily and quickly. After recovery from anesthesia, they were returned to the feeding room. Forty-eight hours after the first SAH, a second one was produced in the same manner as the first. In control animals, the same technique was applied with injection of sterile saline instead of blood.

### 2.3. Experimental Design

The experimental groups consisted of control group (*n* = 12), SAH group (*n* = 12), SAH + vehicle group (*n* = 12), and SAH + melatonin (*n* = 12). In the animals of SAH + melatonin group, melatonin (5 mg/kg) was administered immediately after first blood injection and was continued every 12 hours for 120 hours. Rabbits of SAH + vehicle group received equal volumes of vehicle (1% ethanol in 1 mL saline) at corresponding time points. Both melatonin and vehicle were administered intraperitoneally. Melatonin (Sigma, St Louis, MO, USA) was dissolved in absolute ethanol and further dilutions were made in saline to reach a final concentration of 1% (v/v) ethanol. The dose was chosen according to Aydin and coworkers since they observed beneficial effects on reducing the vasoconstriction of the basilar artery following SAH after treatment with melatonin using the same dose [5]. All the rabbits were killed on day 5.

Six rabbits in each group were sacrificed with the fixation-perfusion method. The basilar arteries were taken for hematoxylin and eosin (H&E) staining. The other six rabbits in each group were exsanguinated and decollated. The artery was removed and rinsed in 0.9% normal saline (4°C) several times to wash away blood and blood clot. And then the artery was frozen in liquid nitrogen immediately for biochemical studies.

### 2.4. Perfusion-Fixation

The rabbits scheduled for death were anesthetized with an intramuscular injection of a mixture of ketamine (40 mg/kg) and droperidol (2.5 mg/kg). The animals were then intubated endotracheally with a 3.5 mm diameter tracheal tube and mechanically ventilated with a rodent ventilator (SGC, China). Perfusion-fixation was then performed. The thorax was opened with a cannula placed in the left ventricle, the descending thoracic aorta clamped, and the right atrium open. Perfusion was begun with 500 mL of physiological phosphate buffer solution (PBS, pH 7.4) at 37°C, followed by 500 mL of 10% buffered formaldehyde under a perfusion pressure of 120 cm H_2_O. After perfusion-fixation, the whole brain with the basilar artery was removed and immersed in the same fixative solution.

### 2.5. Measurement of Blood Vessel Cross-Sectional Area

The degree of cerebral vasospasm was evaluated by the measurement of basilar artery lumen's cross-sectional areas. The formalin-fixed and paraffin-embedded basilar artery sections (4 *μ*m in thickness) were deparaffinized, hydrated, washed, and stained with hematoxylin and eosin. Then micrographs of the basilar arteries were put into the computer. Cross-sectional areas and wall thicknesses of blood vessels were determined by an investigator without knowing the group setting, using the High Definition Medical Image Analysis Program (HMIAP-2000, developed by Tongji Medical University, China). The areas were calculated by measuring the perimeter of the actual vessel lumen and then calculating the area of an equivalent circle (area = *π* *r* ^2^, where *r* = radius) based on the calculated equivalent *r* value from the perimeter measurement (*r* = perimeter/2*π*), thus correcting for vessel deformation and off-transverse sections. For each vessel, three sequential sections (midpoint of the proximal, the middle, and the distal) were taken, measured, and averaged.

### 2.6. Nuclear Protein Extract and Electrophoretic Mobility Shift Assay (EMSA)

Nuclear protein was extracted and quantified as described [12]. EMSA was performed using a commercial kit (Gel Shift Assay System; Promega, Madison, WI) following the methods in our laboratory. The NF-*κ*B oligonucleotide probe (5′-AGTTGAGGGGACTTTCCCAGGC-3′) was end-labeled with [*γ*-^32^P]ATP (Free Biotech, Beijing, China). EMSA was performed according to our previous study [12]. After electrophoresis, the gel was transferred to Whatman filter paper, vacuum dried and exposed to Kodak XAR-5 film overnight. Levels of NF-*κ*B DNA binding activity were quantified by scanning the developed Kodak XAR-5 film with a computer-assisted, linear scanning densitometer in transparent mode (Hoefer Scientific Instruments, San Francisco, CA). Data were expressed as arbitrary densitometry units (ADUs) obtained from the densitometric scans.

### 2.7. Enzyme-Linked Immunosorbent Assay (ELISA)

The levels of inflammatory mediators were quantified using specific ELISA kits for rats according to the manufacturers' instructions (TNF-*α* from Diaclone Research, Besançon, France; IL-1*β*, IL-6 from Biosource Europe SA, Nivelles, Belgium) and our previous study [12]. Values were expressed as ng/g protein.

### 2.8. Measurement of MDA Levels

MDA levels were determined with the method of the previous studies [13, 14]. The principle of the assay depends on the reaction of lipid peroxidation products with thiobarbituric acid and formation of products named as thiobarbituric acid reacting substances, which give maximum absorbance at 532 nm wavelength. Serial dilutions of 1,1,3,3 tetraethoxypropane were used to obtain a standard absorbance versus concentration curve, and MDA concentrations of the tissue samples were determined from this curve. MDA concentrations were given as nmol/g wet tissue.

### 2.9. Measurement of Tissue SOD Activities

SOD enzyme activities were determined with RANSOD (Randox, UK) SOD assay kit. The method of the assay employs xanthine and XOD to generate superoxide radicals which react with INT to form a red formazan dye. The SOD activity is then measured by the degree of inhibition of the reaction. One unit of SOD is that which causes a 50% inhibition of the rate of reduction of INT under the conditions of the assay. SOD activities of the samples were given as U/mg protein.

### 2.10. Measurement of Tissue GSH-Px Activities

GSH-Px Assay (Northwest Life Science Specialities Vancouver, WA, USA) kit was used for the determination of tissue GSH-Px activities and this assay is an adaptation of the method of previous studies [14, 15]. The principle of the assay is as follows: GSH-Px catalyzes the reduction of H_2_O_2_, oxidizing reduced GSH to form GSSG. GSSG is then reduced by GR and *β*-nicotinamide denine dinucleotide phosphate forming NADP+ (resulting in decreased absorbance at 340 nm) and recycling the GSH. Because GSH-Px is limiting, the decrease in absorbance at 340 nm is directly proportional to the GSH-Px concentration. GSH-Px activity is reported as units based on the definition: 1 U of GSH-Px = the amount of enzyme necessary to catalyze the oxidation (by H_2_O_2_) of 1.0 *μ*mol GSH to GSSG per minute at 25°C, pH 7.0. GSH-Px activities of the tissue samples were given as units per milligram of protein.

### 2.11. Statistical Analysis

All data were presented as mean ± SD. SPSS 12.0 was used for statistical analysis of the data. All data were subjected to one-way ANOVA. Differences between experimental groups were determined by the Fisher's LSD posttest. Statistical significance was inferred at *P* < .05.

## 3. Results

### 3.1. General Observation

No significant changes in body weight, mean arterial blood pressure, temperature, or injected arterial blood gas data were detected in any of the experimental groups (data not shown). The rabbits all survived from the procedure of induction of experimental SAH.

### 3.2. Melatonin Administration Ameliorated Cerebral Vasospasm after SAH

**Figure 1:**
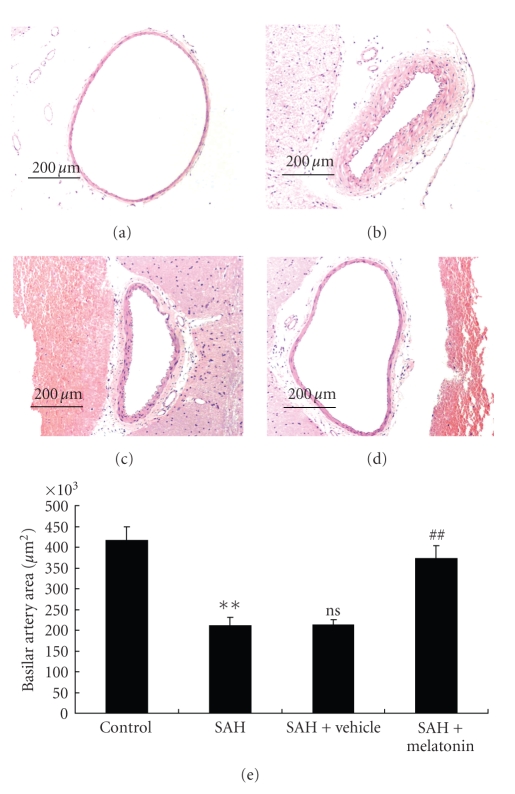
Changes in the cross-sectional area of basilar arteries in the experimental SAH model. (a)–(d) Representative images of cross-sectional areas of the basilar arteries of the control rabbits or rabbits subjected to SAH alone or SAH plus injection with vehicle or melatonin. Severe vasospasm could be detected in the SAH group, which was attenuated in the SAH + melatonin group. (a) The control group; (b) the SAH group; (c) the SAH + vehicle group; (d) the SAH + melatonin group; (e): Histogram of the average cross-sectional area of the basilar arteries from different groups. There is a significant difference in the basilar artery cross-sectional area between the SAH and control groups. The basilar artery cross-sectional area was significantly increased in the SAH + melatonin group compared with the SAH or SAH + vehicle groups. Results are represented as means ± SD of six rabbits in each group. **P < .01 versus control group, ns *P* > .05 versus SAH group; ^##^ *P* < .05 versus SAH + vehicle group.

As shown in Figure 1, there was a significant difference in the cross-sectional area of basilar artery among all the groups on day 5 following SAH (*P* < .01). A significant difference was detected between the SAH (211745.2 ± 19158.4 *μ*m^2^) and the control (416253.4 ± 32508.4 *μ*m^2^) groups (*P* < .01) (Figure 1). There was also a significant difference in the basilar arterial cross-sectional area between the SAH + melatonin (372806.5 ± 31195.3 *μ*m^2^) and SAH + vehicle (213345.6 ± 12213.5 *μ*m^2^) groups (*P* < .01) (Figure 1). No significant difference was seen between the SAH group and the SAH + vehicle group (*P* > .05).

### 3.3. Effects of Melatonin on NF-*κ*B Binding Activity in SAH Basilar Arteries

**Figure 2:**
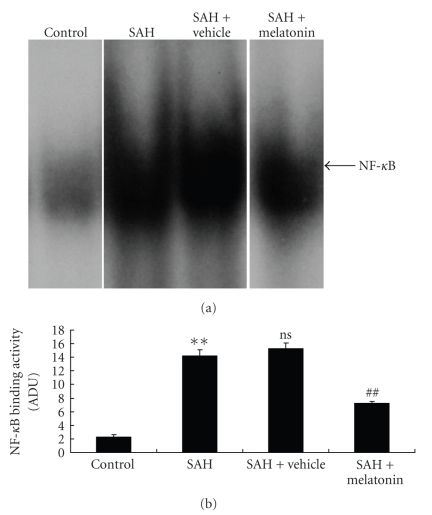
NF-*κ*B activity in the basilar arteries in control group (*n* = 6), SAH group (*n* = 6), SAH + vehicle group (*n* = 6), and SAH + melatonin group (*n* = 6). (a) EMSA autoradiography of NF-*κ*B DNA binding following SAH. (b) Levels of NF-*κ*B DNA binding activity quantified by computer-assisted densitometric scanning and expressed as an arbitrary densitometric units (ADUs). The activity of NF-*κ*B DNA binding was significantly increased in the animals of SAH and SAH + vehicle groups compared with the control group (*P* < .01). Compared to SAH or SAH + vehicle group, melatonin significantly suppressed NF-*κ*B activation in SAH + vehicle group (*P* < .01). Bars represent the mean ± SD. **P < .01 compared with control group, ns *P* > .05 compared with SAH group; ^##^ *P* < .01 compared with SAH + vehicle group.

To determine the influence of melatonin on NF-*κ*B binding activity in the basilar arteries post SAH, EMSA was performed to detect the changes of NF-*κ*B as described in Section 2. EMSA autoradiography of NF-*κ*B DNA binding activity of in the basilar arteries was shown in Figure 2. Low NF-*κ*B binding activity (weak EMSA autoradiography) was found in the control group. Compared with the control group, NF-*κ*B binding activity in the artery was significantly increased (*P* < .01) in SAH or SAH + vehicle group on day 5 after SAH. There was no statistically significant difference between SAH group and SAH + vehicle group (*P* > .05) (Figure 2). In SAH + melatonin group, arterial NF-*κ*B binding activity was significantly downregulated (*P* < .01) after melatonin injections.

### 3.4. Melatonin Treatment Decreased Vascular Levels of Proinflammatory Cytokines following SAH

**Figure 3:**
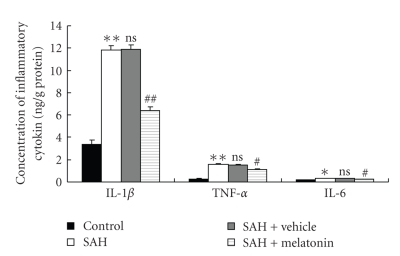
Changes of inflammatory mediators in the basilar arteries as determined by ELISA in control group (*n* = 6), SAH group (*n* = 6), SAH + vehicle group (*n* = 6), and SAH + melatonin group (*n* = 6). SAH could induce the significantly increased concentrations of IL-1*β*, TNF-*α*, and IL-6 in rabbit vascular tissue. In SAH + melatonin group, the arterial concentrations of IL-1*β*, TNF-*α*, and IL-6 were markedly downregulated as compared with that of SAH or SAH + vehicle group. *P < .05 and **P < .01 versus Control group, ns *P* > .05 versus SAH group, ^#^ *P* < .05 and ^##^ *P* < .01 versus SAH + vehicle group.

Concentrations of IL-1*β*, TNF-*α*, and IL-6 were low in the basilar arteries of the control group (3.35 ± 0.43, 0.28 ± 0.07, and 0.18 ± 0.01 ng/g protein, resp.) (Figure 3). Compared with Control group, vascular levels of the three inflammatory cytokines were greatly induced in SAH or SAH + vehicle group. As shown in Figure 3, melatonin administration after SAH could lead to significantly decreased IL-1*β*, TNF-*α* and IL-6 concentrations. The result showed that melatonin administration following SAH could downregulate the expressions of Proinflammatory cytokines in the basilar arteries.

### 3.5. Influence of Melatonin on Oxidative Stress in the Basilar Arteries after SAH

The values of the tissue MDA levels and tissue SOD and GSH-Px enzyme activities were shown in Table 1. SAH significantly increased the tissue MDA levels (*P* < .05) and significantly decreased the tissue SOD and GSH-Px enzyme activities (*P* < .05) when compared with controls. Melatonin treatment has shown protective effect via decreasing significantly (*P* < .05) the elevated MDA levels and also significantly increasing the reduced antioxidant enzyme activities (SOD, *P* < .01; GSH-Px, *P* < .05).

## 4. Discussion

The main findings of this study are that (1) vascular inflammatory mediators were induced after SAH and could be remarkably repressed when treated with melatonin; (2) the increased lipid peroxidation in the artery tissues could be significantly downregulated after melatonin injections following experimental SAH; (3) after melatonin administration, the postSAH reduced antioxidative status was ameliorated in this two-hemorrhage model; (4) in agreement with the previous research [5], treatment with melatonin prevented cerebral vasospasm of the basilar arteries in rabbits. These findings suggest for the first time that melatonin could regulate vascular inflammation and oxidative stress, and this may be one mechanism by which melatonin attenuates the development of cerebral vasospasm after SAH.

There have been many studies focusing on the neurovascular protective effects of melatonin in SAH models [5–7, 16]. Most of these previous studies used the one-hemorrhage SAH model; however in the current research, we used the two-hemorrhage model that was more suitable for SAH-cerebral vasospasm research than one-hemorrhage model because the time course of vasospasm accurately correlates with that observed in humans, which peaked on day 5 after SAH [17]. Thus, in the present study, we chose day 5 as the research time-point. Also, we gave melatonin after the onset of hemorrhage, which matches the clinical setting.

In the circumstance of SAH, a complex series of cellular and molecular events is elicited by the presence of a blood clot in the subarachnoid space, culminating in a robust inflammatory response. Following cellular and molecular events were involved: (1) expressions of adhesion molecules that evoke leukocytes adhesion to the endothelium; (2) cytokines production; (3) immunoglobulin and complement activation. Cerebral vasospasm might result from the interaction among those events [8]. During the whole physiopathologic process of cerebral vasospasm, NF-*κ*B and Proinflammatory cytokines played important roles [9].

In the research regarding melatonin and inflammatory mediators, Alonso et al. investigated the effects of melatonin on the expressions of inducible nitric oxide synthase (iNOS) and NF-*κ*B in rat skeletal muscle after acute exercise [18]. Their results indicated that melatonin had potent protective effects against damage caused by acute exercise in rat muscle, preventing oxidative stress, NF-*κ*B activation, and iNOS over-expression. At the same time, the results from another previous study showed that melatonin exhibited a superior capacity to reduce the Proinflammatory cytokines (IL-1*β*, IL-6, and TNF-*α*) in amyloid-beta-induced oxidative stress model [19]. However, data was still scarce concerning the effects of melatonin on the vascular inflammation in SAH models. In the present study, we confirmed that SAH could activate the inflammatory mediators such as NF-*κ*B, IL-1*β*, IL-6, and TNF-*α*, which has been reported in the previous studies [8, 9]. Furthermore, we put forward that melatonin treatment could suppress the vascular inflammatory response in this rabbit SAH model.

Excessive production of free radicals and subsequent lipid peroxidation has been suggested causally related to cerebral vasospasm after SAH [6, 20]. There are several sources for the excessive generation of free radicals following SAH, including disrupted mitochondrial respiration and extracellular hemoglobin but the principal origin in vasospasm is thought to be the autooxidation of oxyhemoglobin to methemoglobin, which leads to production of superoxide radical [6]. Antioxidant agents such as 1,2-bis(nicotinamide)-propane, tirilazad, resveratrol, and ebselen have been shown to ameliorate experimental vasospasm and reduce lipid peroxidation in some different animal models [6]. upregulation of free radical producing enzymes and inhibition of intrinsic antioxidant systems such as SOD and GSH-Px occurs after SAH and leads the brain to oxidative damage [6]. As revealed by Ersahin [7], treatment with melatonin due to its free radical scavenging properties significantly inhibited SAH-induced lipid peroxidation and neutrophil infiltration of the brain tissue on the second day of SAH induction, while the depleted antioxidant GSH level and inhibited Na^+^-K^+^-ATPase activity were restored to control levels. However, for the vascular tissues, the effect of melatonin on oxidative stress following SAH still remains unknown. Our data demonstrated that treatment with melatonin induced the antioxidant defense system, downregulated lipid peroxidation in the basilar artery, and resulted in a significant increase of the cross-sectional area of basilar artery after SAH. Therefore, our results suggested the possibility of a vascular oxidation preventive function of melatonin administration following experimental SAH.

In summary, to the best of our knowledge, this is the first study to demonstrate the effects of melatonin on inflammation and oxidative stress in the cerebral artery after SAH. We found that SAH could upregulate the inflammatory mediators, increase lipid peroxidation, and inhibit the antioxidant defense system in the rabbit basilar artery, which could be markedly modulated by melatonin administration. The treatment of melatonin in this SAH model resulted in attenuation the degree of cerebral vasospasm following SAH. These results suggest that SAH could induce vascular inflammatory process and oxidative stress in the arterial wall which might play a central role in the pathogenesis of cerebral vasospasm. The therapeutic benefit of postSAH melatonin administration might be due to its salutary effect on modulating Proinflammatory mediators and biomarkers of oxidative stress.

## Figures and Tables

**Table 1:** Vascular antioxidant status of the experimental group of animals.

Groups	*n*	MDA (nmol/g)	SOD (U/mg)	GSH-Px (U/mg)
Control	6	31.28 ± 8.27	0.52 ± 0.1	1.08 ± 0.21
SAH	6	48.12 ± 6.13*	0.21 ± 0.08*	0.72 ± 0.16*
SAH + vehicle	6	46.21 ± 5.07^ns^	0.22 ± 0.11^ns^	0.76 ± 0.08^ns^
SAH + melatonin	6	38.07 ± 7.1^#^	0.43 ± 0.12^##^	1.02 ± 0.31^#^

In the table, arterial MDA concentrations were given per gram of wet tissue; SOD, GSH-Px enzymatic activities were given per mg/protein. Values are expressed as mean ± SD. (*P < .05 and **P < .01 versus Control group, ns *P* > .05 versus SAH group, ^#^ *P* < .05 and ^##^ *P* < .01 versus SAH + vehicle group).
